# Laparoscopic low anterior resection with en bloc total hysterectomy and bilateral salpingectomy for stage IV endometriosis—A video vignette

**DOI:** 10.1111/codi.70512

**Published:** 2026-06-04

**Authors:** Maher Al Khaldi, Vanessa Fuentes, Alison Schneider, Pamela Frazzini Padilla, Giovanna Da Silva

**Affiliations:** ^1^ Ellen Leifer Shulman and Steven Shulman Digestive Disease Center Cleveland Clinic Florida Weston Florida USA; ^2^ Department of Minimally Invasive Gynecology Surgery Cleveland Clinic Florida Weston Florida USA

Stage IV endometriosis with rectal involvement can closely mimic malignancy and requires coordinated multidisciplinary management. We present a 45‐year‐old female with diarrhoea and rectal bleeding found on colonoscopy to have a partially obstructing mid‐rectal mass. Biopsy was benign, CEA was normal and pelvic MRI demonstrated a 5.8‐cm deep infiltrative endometriotic lesion invading the anterior rectal wall. A combined gynaecologic and colorectal laparoscopic approach was undertaken. Prophylactic bilateral ureteral catheterization with indocyanine green facilitated ureteral identification during extensive ureterolysis for retroperitoneal fibrosis. Total hysterectomy with bilateral salpingectomy and ovarian preservation was performed, followed by meticulous rectal mobilization and low anterior resection. The specimen was extracted transvaginally en bloc. A tension‐free stapled colorectal anastomosis was constructed with negative leak testing and confirmed perfusion on fluorescence angiography. The patient recovered uneventfully and was discharged on postoperative day three.

Management of stage IV endometriosis involving the rectum should be individualized and multidisciplinary. When deep infiltrative disease causes obstruction, bleeding or significant symptoms, en bloc resection with restoration of bowel continuity can provide definitive symptom relief while preserving organ function in carefully selected patients (Video 1).

**VIDEO 1:** The endometrioma densely tethered to the rectum, uterine cervix and left ureter. Video content can be viewed at https://onlinelibrary.wiley.com/doi/10.1111/codi.70512.

## AUTHOR CONTRIBUTIONS

**Maher Al Khaldi:** Conceptualization; visualization; writing – original draft; data curation. **Vanessa Fuentes:** Writing – review and editing; conceptualization; visualization; data curation. **Alison Schneider:** Conceptualization; writing – review and editing; visualization; data curation. **Pamela Frazzini Padilla:** Conceptualization; visualization; writing – review and editing; data curation. **Giovanna Da Silva:** Conceptualization; visualization; writing – review and editing; project administration.

## FUNDING INFORMATION

None.

## CONFLICT OF INTEREST STATEMENT

The authors declare no conflicts of interest.

## ETHICS STATEMENT

This video recording was deemed exempt from ethics approval as the patient gave informed consent.

## Data Availability

Upon reasonable request from the first author at alkhalm@ccf.org.

